# PReS-FINAL-2121: Results for 6 minute walk values in healthy German children show similar results as from Britain

**DOI:** 10.1186/1546-0096-11-S2-P133

**Published:** 2013-12-05

**Authors:** I Foeldvari

**Affiliations:** 1 Hamburger Zentrum für Kinder- und Jugendhreumatologie, Hamburg, Germany

## Introduction

6 minute walk is a primary outcome measure in therapeutic studies for patients with pulmonary hypertension. Currently we have a two of sets of data[1,2] regarding test results in the 6 minute walk test (6MWT) in healthy children with a large span in the norm values in the different age groups.

## Objectives

Aim of the study was to establish norm values for healthy German children for the 6 Minute Walk Test.

## Methods

The team of an occupational therapist and a study nurse were visiting schools. Permission from the parents was give before the test. Always just probands from one class were invited to participate. The test were performed according the international guidelines[3]. The demographic data of the probands were collected and the parents filled out a short survey regarding the physical activity and the health condition. Children with chronic diseases, which decrease the stamina were excluded.

## Results

Up till now 611probands participated from the age 5 ot 14 years. 343 of the 611 were female. The mean 6 minute walk continuously increased with age (Table 1.). It correlated in the age groups with the BMI.

**Table 1:** 

6 ( n = 21)	491.6	479.6	6 (n = 18)	472.2	481.1
7 (n = 42)	494.2	487.2	7(n = 26)	487.9	476.9

8 (n = 43)	485.0	494.8	8 (n = 33)	488.2	493.8

9 ( n = 31)	512,0	520.1	9 ( n = 49)	492.0	504.5

10 (n = 48)	516.8	519.7	10 ( n = 58)	526.3	521.3

## Conclusion

Our results are in the range of the patients from the UK published by Lammers et al [1] and are in significantly lower range than in the Chinese population collected data by Li et al.[2]. This reflects the importance of this study to gain norm values for our patient population.

## Disclosure of interest

None declared.
